# Factors associated with essential newborn care practice among obstetric care providers in public hospitals in Gamo, Gofa, and Wolayta zones, southern Ethiopia: A facility-based cross-sectional study, 2022

**DOI:** 10.1371/journal.pone.0314767

**Published:** 2024-12-27

**Authors:** Samuel Shanko Salo, Eshetu Yisihak Ukumo, Manaye Yihune Teshale

**Affiliations:** 1 Department of Midwifery, College of Medicine and Health sciences, Arba Minch University, Arba Minch, Ethiopia; 2 School of Public Health, College of Medicine and Health sciences, Arba Minch University, Arba Minch, Ethiopia; 3 Department of Health Promotion, CAPHRI Care and Public Health Research Institute, Maastricht University, Maastricht, Netherlands; Mizan-Tepi University, ETHIOPIA

## Abstract

**Background:**

Almost everywhere, neonatal mortality can be decreased with ease if competent obstetricians give the necessary treatment. Unfortunately, observational techniques were not used to examine basic essential newborn care practice among obstetric care providers in Ethiopia. Thus, the purpose of this study was to evaluate factors related to essential newborn care practice using observational techniques among obstetric care providers in public hospitals in the Gamo, Gofa, and Wolayta zones, southern Ethiopia.

**Methods:**

An institutional-based cross-sectional study carried out from May 15 to June 30, 2022. A simple random sampling method was used. Structured self-administered questionnaires with a clinical observational checklist were used to collect data. Data imported into Epidata version 4.6 and analyzed using the SPSS Version 25. Bivariable and multivariable analyses were used to identify factors associated with essential newborn care practices. An odds ratio with a 95% confidence interval was used to assess the direction and strength of the association.

**Results:**

The overall magnitude of good essential newborn care practice among obstetric care providers was 53.5% (95% CI = 49, 58). Factors positively associated with the practice of essential newborn care were having interest on working in delivery room (AOR = 3.16, 95% CI = 1.71,5.83), having no work load (AOR = 2.96, 95% CI = 1.78,4.49), received in-service training (AOR = 3.09, 95% CI = 1.75,5.45), having supportive supervision (AOR = 3.41, 95% CI = 1.25, 9.24), and having good knowledge on essential newborn care (AOR = 3.04, 95% CI = 1.89,4.90).

**Conclusion:**

The observed level of essential newborn care practices among obstetric care providers underscores the necessity for targeted interventions that stimulate interest in delivery room work, effectively manage workloads, and offer comprehensive training along with supportive supervision. By concentrating on these aspects and enhancing providers’ knowledge, we can significantly improve essential newborn care practices.

## Introduction

Essential newborn care (ENC) is basic care given to all newborn babies in the delivery room by skilled personnel during their first hours of life to support their survival and wellbeing [1]. It includes drying and stimulating, assessing breathing, cord care, skin-to-skin contact, initiating exclusive breastfeeding, eye care, vitamin K provision, placing an identification band on the baby, measuring the baby’s vital signs, including temperature, and weighing [1, 2]. The newborn’s capacity to adjust to life outside the womb is crucial in the first few hours following delivery [3], and obstetric care providers play a crucial role during this time [4].

Neonatal mortality can quickly and easily be reduced in virtually every part of the world, even in low-income countries, if safe childbirth and effective and ENC are practiced by skilled obstetric care providers [5]. However, due to the poor practice of ENC among a considerable number of obstetric care providers, the majority of neonates delivered in hospitals receive insufficient treatment [6].

According to previous research, the magnitude of good ENC practice among obstetric care providers varies in different parts. A study conducted in the central region of Vietnam showed a magnitude of 53.2% [7]. In Africa, it is about 41.1% in the Khartoum state teaching hospitals [8] and 62.9% in Nigeria [9]. In Ethiopia, it ranges from 24% in rural Gedeo zone health facilities [10] to 74.8% in South Gondar zone health facilities [11], indicating that the magnitude of ENC practice is poor.

In most of the studies, the known factors stated for the poor practice of essential newborn care among obstetrics care providers were: work experience of fewer than 5 years; inadequate knowledge of ENC; having a workload; having no interest in providing essential newborn care [12–14]; unavailability of adequate materials like guidelines and drugs; having a diploma educational status [11].

Children who die within the first 28 days of life often suffer from various illnesses and issues due to inadequate ENC provided at birth [15]. The majority of these are births that are premature and underweight, hypoxia at delivery, hypoglycemia, and infections [5]. Globally, approximately half of neonatal fatalities occur on the day of birth due to a lack of sufficient care during birth [15], which makes the neonatal period responsible for the highest rate of death of any period in childhood [16]. According to the United Nations (UN) inter-agency group on child mortality estimation report, more than 60 nations would miss the target for newborn mortality (12 or fewer deaths per 1,000 live births) by 2030 if the current trend continues [17]. In Ethiopia, the 2019 Mini Ethiopia Demographic and Health Surveys (MEDHS) report shows the neonatal mortality rate has increased to 33 per 1,000 live births [18], up from 29 per 1,000 live births in the 2016 EDHS [19], thus indicating that we are on the wrong track to meet the Sustainable Development Goal by 2030 (12 or fewer deaths per 1,000 live births).

Evidence suggests that the majority of these fatality rates, particularly those that occur after birth and shortly after, can be prevented by using simple, evidence-based fundamental newborn care practices carried out by experienced obstetric care providers with readily available facilities [20]. Additionally, the essential care for every baby programme aims to equip providers with the knowledge and skills to provide the majority of ENC and to assist mothers and families in providing this care [21]. Integrated management of newborn and childhood illness (IMNCI) 2021 also incorporated measuring baby vital signs in their chart booklet for health care providers as an additional component of ENC [2].

To find the practice gaps, several investigations on the degree of essential newborn care practice among obstetric care providers were carried out in Ethiopia [11, 13, 20, 22–24]. However, using observational data and a standard checklist that includes newborn vital signs, little is known about the practice of providing ENC and its associated factors among obstetric care providers.

Dealing with the factors affecting ENC practice among obstetric care providers is very vital for health facilities and other stakeholders to give direction for planning and designing strategies to decrease neonatal mortality in the Ethiopian context. Also, it will help newborns get quality ENC by narrowing evidence gaps and providing fruitful findings for responsible bodies to avoid associated factors of ENC practice among obstetric care providers. That is why this study was aimed at assessing factors associated with essential newborn care practice among obstetric care providers in the public hospitals of Gamo, Gofa, and Wolayta zones of southern Ethiopia by using observational data with a standard checklist.

## Materials and methods

### Study setting, design and period

The study was conducted in public hospitals in the Gamo, Gofa, and Wolayta zones of Southern Ethiopia. Gamo, Gofa, and Wolayta zones are the three zones out of eleven zones in the south Nations Nationalities and Peoples Republic (SNNPR), with the administrative towns of Arba Minch, Sawula, and Soddo, respectively.

The Gamo, Gofa, and Wolayta zones have a total number of sixteen (16) public hospitals at primary, general, and referral levels, containing 457 obstetric care providers who provide essential newborn care.

Specifically, the Gamo has six public hospitals, namely: Arba Minch general hospital, Dilfana primary hospital, Chencha primary hospital, Gerese primary hospital, Kamba primary hospital, and Selam Ber primary hospital, with 165 obstetric care providers. Gofa zone has two public hospitals, namely, Sawula general hospital and Laha primary hospital, with 48 obstetric care providers, and Wolayta zone has a total of eight public hospitals, namely: Wolayta Soddo University teaching, referral, and comprehensive specialized hospital; Bale primary hospital; Bodity primary hospital; Bitana primary hospital; Humbo primary hospital; Halale primary hospital; Gasuba primary hospital; and Bombe primary hospital with 244 obstetric care providers. A facility-based cross-sectional study design was conducted in these hospitals from May 15 to June 30, 2022.

### Participant selection and exclusion

**Fig 1:**
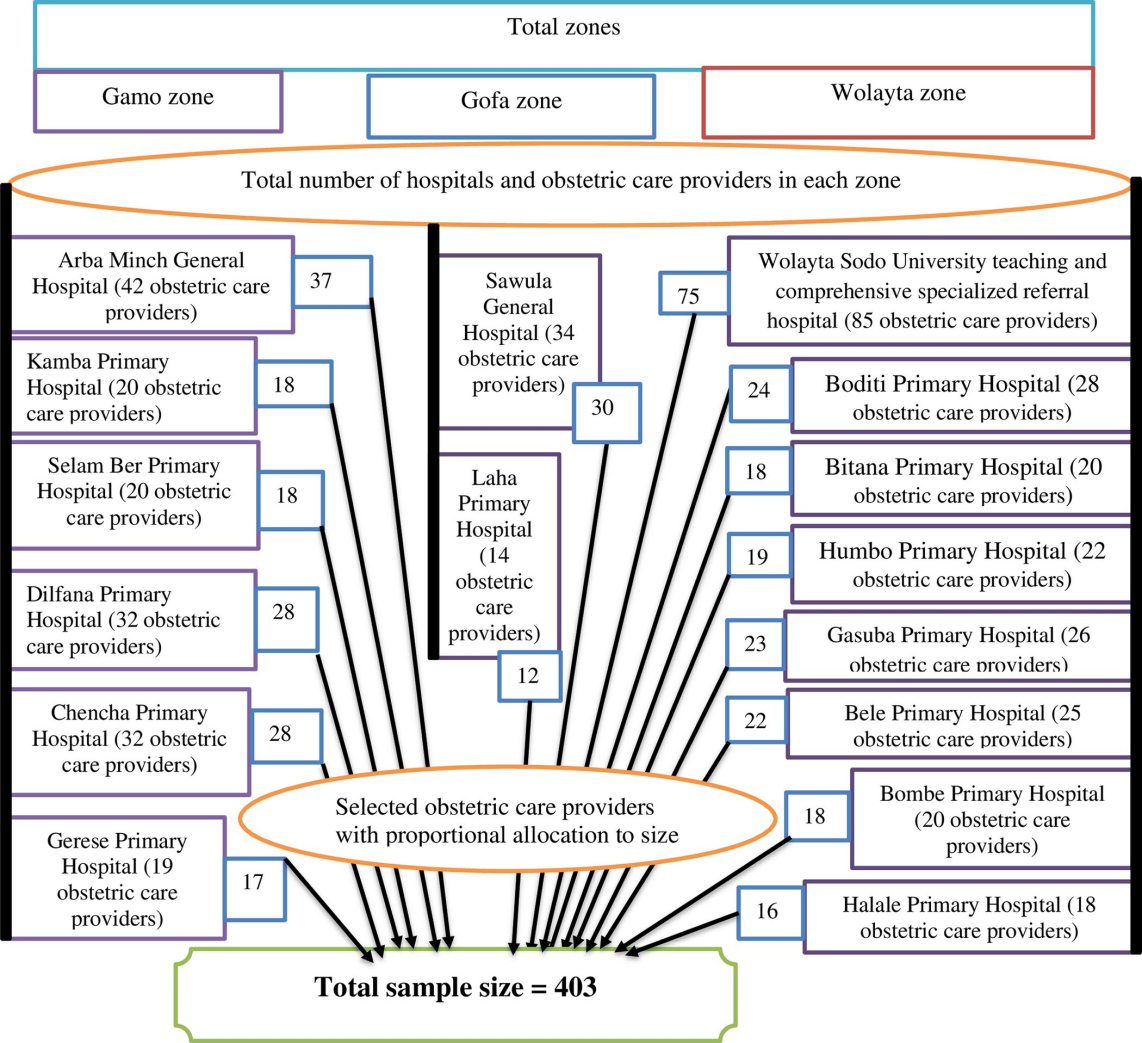
Schematic presentation of sampling procedure to select study participants from each public hospital of Gamo, Gofa, and Wolayta zones, southern Ethiopia, 2022.

All obstetric care providers working in the maternal, newborn, and child health (MCH) units of Gamo, Gofa, and Wolayta zone public hospitals were the source populations, and from those, all obstetric care providers who were assigned to and working in the labor and delivery wards of these three zones public hospitals were selected as the study population (Fig 1).

However, obstetric care providers who were out of the ward during the data collection period for different reasons like training, annual leave, and maternity leave were excluded.

### Sample size determination and sampling procedure

The sample size required for the study was calculated using the single population proportion formula with the following assumptions: The prevalence of the overall practice of ENC was 52.4%, taken from a study conducted in central zone health facilities in Tigray [23] with a confidence interval of 95% and a margin of error of 5%. By considering 5% of none-response rate, the final sample size was 403.

A simple random sampling technique was applied to select study participants from all 16 public hospitals that provide ENC service in Gamo, Gofa, and Wolayta zones. All public hospitals in these three zones were included. The study participants were allocated proportionally to each zonal public hospital (Fig 1). Computer-generated random sampling was applied based on their programme list to select study participants.

### Data collection methods and procedures

Sixteen trained BSc midwives collected data from the participants using a self-administered questionnaire with four different sections, including: socio-demographic characteristics; personal-related; institutional-related; knowledge on specific ENC; and a clinical observational checklist, including measuring baby vital signs.

Knowledge on specific ENC-related questions was comprised of 21 items with multiple choices. The response was counted as 0 or 1 for a single question. An observational checklist was copied for three observations, containing 19 items with 1 and 0 coding for a single participant to observe on three different neonates to say whether the task was performed or not. The sum score from three observations was used to determine the good or poor practice of ENC.

### Study variables

The dependent variable of this study was essential newborn care practice. Independent variables were socio-demographic factors like age, sex, marital status, profession (field of study), qualification, work experience, and monthly salary. Personal-related factors like: interest in working in the delivery room, work experience with delivery services, manageable work load, in-service training on essential newborn care, frequency of in-service training, supportive supervision, and knowledge of specific ENC. Institutional-related factors like type of facility, availability of training guidelines, availability of cord tie, availability of suction device, availability of vitamin K injection, availability of baby identification material, and availability of TTC eye ointment.

### Operational definitions and measurements

#### Essential newborn care

Is provided to a newborn immediately after delivery and includes: drying the baby’s body with a dry and warm towel; stimulating breathing while drying; wrapping with another dry towel and covering the head while the baby is on the mother’s abdomen; evaluating breathing while drying and managing accordingly; clamp or tie the cord; place the baby in skin-to-skin contact with the mother; initiate breastfeeding immediately within the first hour of life; apply eye ointment once on both eyes; apply chlorhexidine on the cord; administer vitamin K (1 mg for a term or 0.5 mg for a preterm baby) intramuscularly on the anterior mid-thigh; measure the baby’s vital signs, weigh the newborn, classify, and record all documentation on the patient’s card and registration book [2, 14].

#### Manageable workload

When the ratio of the obstetric care provider to the client is less than one [13].

#### Good knowledge

An obstetric care provider who scores 75% or above on knowledge-related questions about specific newborn care is said to be knowledgeable or have good knowledge [24].

#### Poor knowledge

An obstetric care provider that scores below 75% of knowledge-related questions about specific newborn care is said to have poor knowledge [24].

#### Essential newborn care practice

Participants were considered to have good practice if they correctly performed at least 70% or more of the tasks in the checklists based on the three observations. If they perform below 70% of the tasks in the checklist on the three observations considered as having poor practice [6].

#### Obstetric care provider

An accredited health professional such as a midwife, doctor, or health worker who has been educated and trained to proficiency in the skills needed to manage normal (uncomplicated) pregnancies, childbirth, and the immediate postnatal period, and in the identification, management, and referral of complications in women and newborns [24].

### Data quality control

In order not to miss an important idea, the questionnaire was prepared in English and translated into Amharic, then back-translated to English for analysis. A pre-test was done on 8% of participants at Nigist Ellini Comprehensive and Specialized Hospital (Wachamo), and the necessary modifications were made based on the results. Intensive one-day training was given for data collectors. The data collection process was supervised on a daily basis, and finally, the collected data were checked for consistency, omissions, and completeness of the questions.

### Data processing and analysis

The collected data were coded, cleaned, and entered into Epi Data version 4.6, which was then exported to SPSS version 25 for further management and analysis. Descriptive statistics were carried out. Binary logistic regression was used to assess the association between the dependent variable and independent variables. The variables with a P-value ≤0.25 in binary logistic regression were fitted to multivariable logistic regression. A multivariable logistic regression with the likelihood ratio method was fitted to identify independently associated factors. An adjusted odds ratio (AOR) with a 95% confidence interval (CI) and corresponding P-value were used to identify statistically significant factors. A P-value <0.05 was used to declare statistical significance. Multi-collinearity was checked, showing the highest variable inflation factor (VIF) of 2.94 (tolerance = 0.33), indicating no multi-collinearity. The Hosmer-Lemeshow goodness of fit test confirmed model fitness (p-value = 0.49).

### Ethical approval and consent to participate

The Arba Minch University institutional ethical review board approved the study with reference number IRB/1271/2022, and authorization letters were acquired from all zonal hospitals and health departments. After providing participants with a thorough explanation of the study’s objectives, volunteers provided written consent to confirm their willingness to participate. It was made clear to participants that they could leave the research at any point while completing the self-administered questionnaire.

## Results

### Socio-demographic characteristics of the respondents

Out of the total of 403 obstetric care providers, 396 participated in our study (with a response rate of 98.26%). Of those, 250 (63.1%) were female. More than half of them, 222 (56.1%), are between the ages of 26 and 30. Two hundred twenty-three (56.3%) participants were married. From all participants, the majority, 362 (91.4%), were midwives, 259 (65.4%) had a degree or higher in their qualification, and 237 (59.8%) had work experience lasting more than three years (Table 1).

**Table 1:** Socio-demographic characteristics of obstetric care providers in the public hospitals of Gamo, Gofa and Wolayta zones, southern Ethiopia, 2022 (N = 396).

Variables	Category	Frequency (N)	Percent (%)
**Sex**	Male	146	36.9
	Female	250	63.1
**Age in year**	21–25	65	16.4
	26–30	222	56.1
	31–35	86	21.7
	36–40	23	5.8
**Marital status**	Single	166	41.9
	Married	223	56.3
	Widowed	4	1.0
	Divorced	3	0.8
**Religion**	Protestant	196	49.5
	Orthodox	168	42.4
	Muslim	24	6.1
	Catholic	8	2.0
**Profession**	Midwifery	362	91.4
	Other	34	8.6
**Qualification**	Diploma	137	34.6
	Degree and above	259	65.4
**Monthly salary**	2,000–4,999	8	2.0
	5,000–7,999	337	85.1
	8,000–10,999	51	12.9
**Work experience**	<1 year	72	18.2
	1–3 years	87	22
	>3 years	237	59.8

Other = Nurse, Health Officer, Anesthetist or General Practitioner.

### Personal characteristics

Of the obstetric care providers who participated in this study, 314 (79.3%) had an interest in working in delivery rooms, and 220 (55.6%) of participants had no manageable work load. More than half of the participants, 217 (54.8%), received in-service training on essential newborn care. while 359 (90.7%) did not get supportive supervision in the last three months (Table 2).

**Table 2:** Personal characteristics of obstetric care providers in the public hospitals of Gamo, Gofa and Wolayta zones, southern Ethiopia, 2022 (N = 396).

Variables	Category	Frequency (N)	Percent (%)
Interested on working in delivery room	Yes	314	79.3
	No	82	20.7
Work experience of delivery services	<1 year	78	19.7
	1–3 years	101	25.5
	>3 years	217	54.8
Manageable work load	Yes	176	44.4
	No	220	55.6
Receive in-service training on Essential newborn care	Yes	217	54.8
	No	179	45.2
Number of times you got In-service training	1 time	177	81.6
	≥2 times	40	18.4
have supportive supervision with in the last three months	Yes	37	9.3
	No	359	90.7
Number of times you got supportive supervision with in the last three months	1 time	18	48.7
	2 times	14	37.8
	≥3 times	5	13.5

### Institutional characteristics

Regarding institutional characteristics of obstetric care providers, more than half of participants 258 (65.2%), were working in primary hospitals. And majority of participants 330 (83.3%) reported having no difficulty giving ENC due to a lack of training guidelines. All 396 (100%) participants reported having no difficulty giving cord care due to a lack of cord ties. Suction device 395 (99.7%), vitamin K injection 388 (98%), tetracycline eye ointment 324 (81.8%), and term and premature size masks 338 (85.4%) were variables reported as having no difficulty because of their availability in sufficient amounts in selected public hospitals, while baby identification material 293 (74%) was the variable reported as having difficulty due to its absence in selected public hospitals (Table 3).

**Table 3:** Institutional characteristics of obstetric care providers in the public hospitals of Gamo, Gofa and Wolayta zones, southern Ethiopia, 2022 (N = 396).

Variables	Frequency (N)	Percent (%)
Facility type		
Primary hospital	258	65.2
General hospital	65	16.4
Teaching comprehensive and referral hospital	73	18.4
Had difficulty giving ENC due to lack of training guidelines at your facility with in the last three months		
Yes	66	16.7
No	330	83.3
Had difficulty giving a baby’s airway due to a lack of suction device at your facility with in the last three months		
Yes	1	0.3
No	395	99.7
Had missed giving vitamin k due to its absence at your facility with in the last three months		
Yes	8	2
No	388	98
Had missed giving TTC eye ointment due to its absence at your facility with in the last three months		
Yes	72	18.2
No	324	81.8
Had difficulty to put a baby’s identification band due to its absence at your facility with in the last three months		
Yes	293	74
No	103	26
Had difficulty giving a baby’s airway due to a lack of term and premature size masks at your facility with in the last three months		
Yes	58	14.6
No	338	85.4

### Knowledge of obstetric care providers on specific essential newborn care

**Fig 2:**
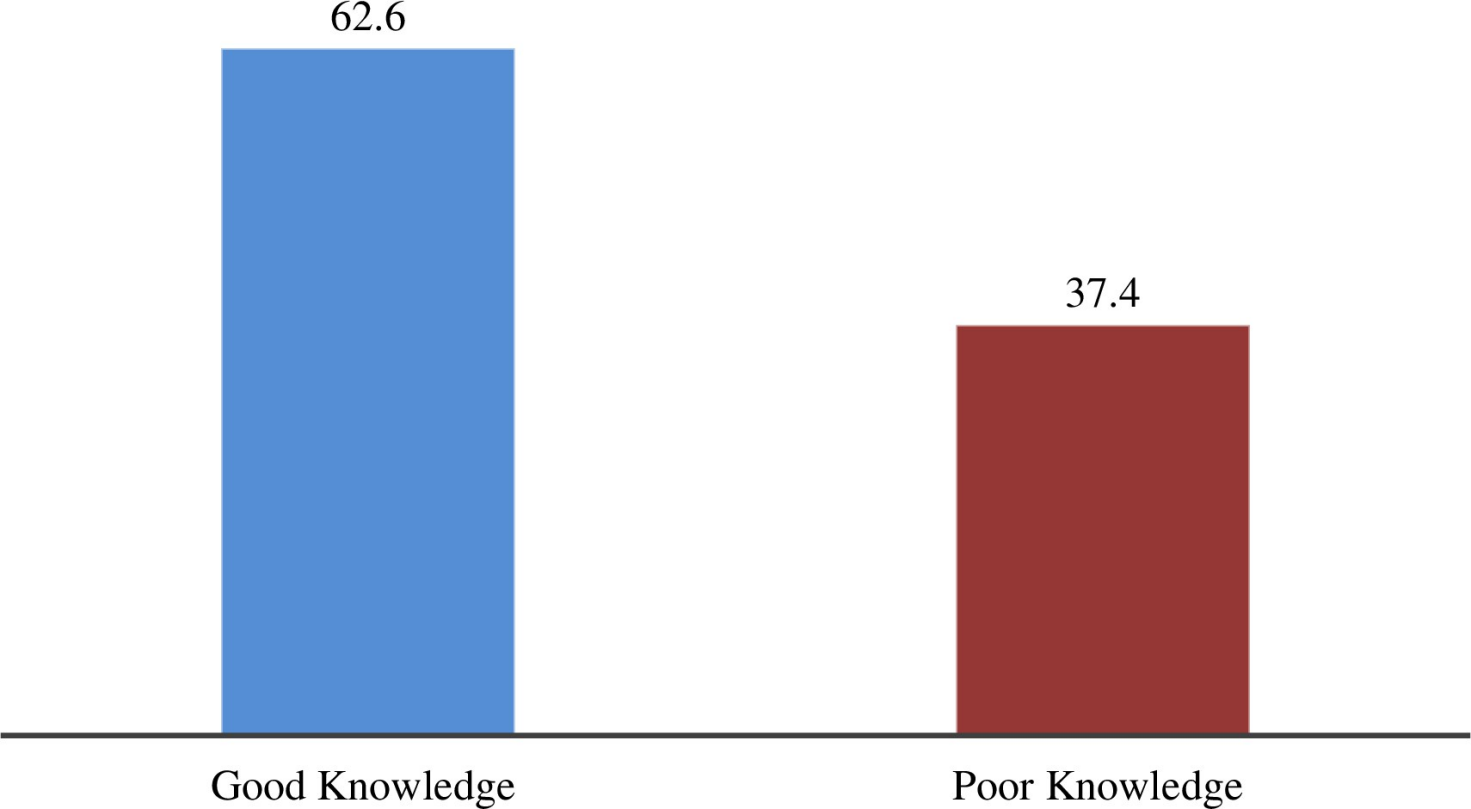
Knowledge status of obstetric care providers of public hospitals in Gamo, Gofa and Wolayta zones, southern Ethiopia, 2022 (N = 396).

Of all the obstetric care providers who participated in this study, 248 (62.6%) had good knowledge about essential newborn care (Fig 2).

### Practice of obstetric care providers on essential new born care

**Fig 3:**
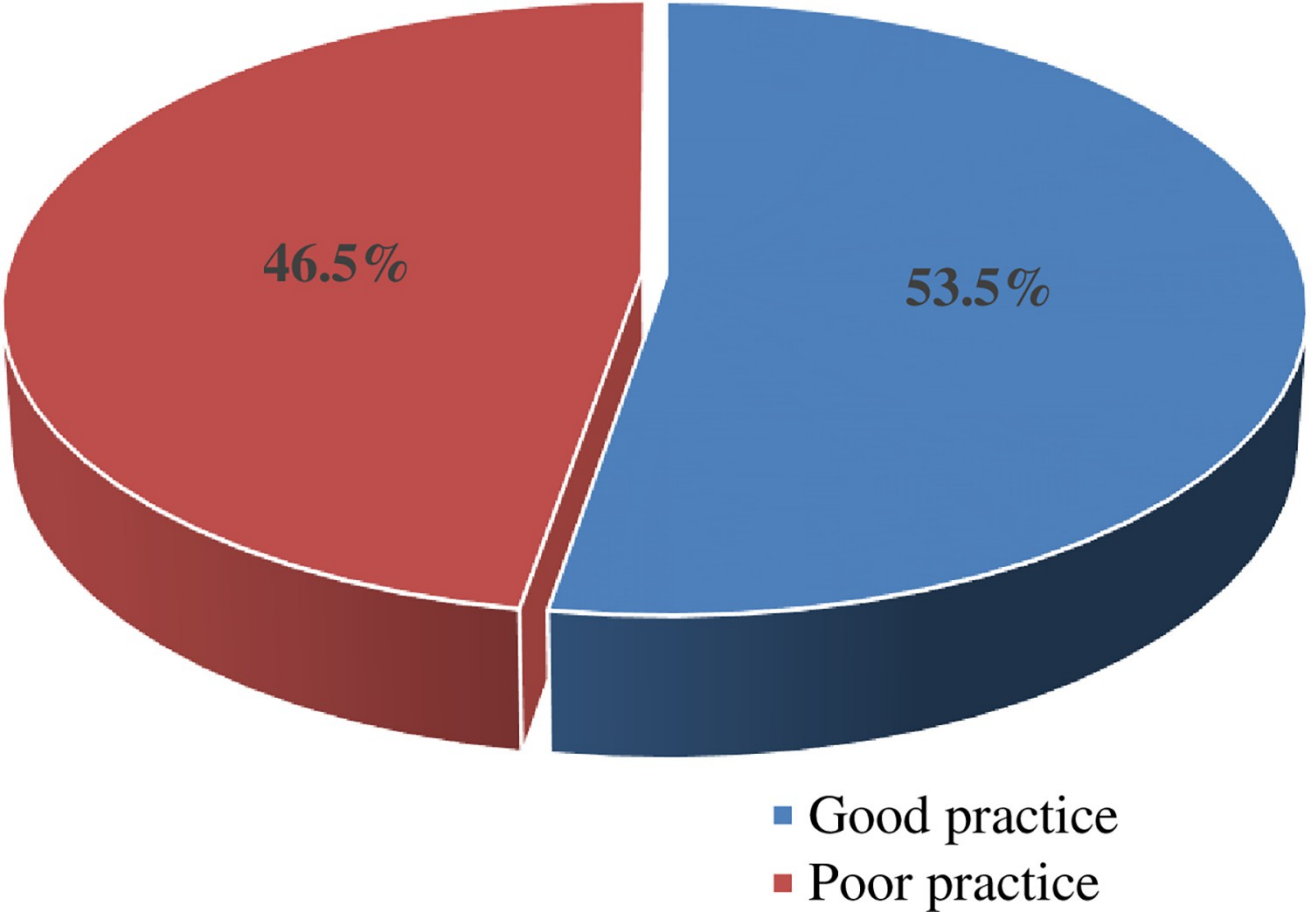
Practice level of obstetric care providers regarding essential newborn care in public hospitals of Gamo, Gofa and Wolayta zones, southern Ethiopia, 2022 (N = 396).

Totally, 396 obstetric care providers were observed while giving care to 1,188 newborns (three times observation) on their level of practice of ENC. The overall good practice of essential newborn care among obstetric care providers was 53.5% (95% CI = 49, 58) (Fig 3).

As three observations of 396 study participants on 1,188 newborns show, 383 (96.7%), 377 (95.2%), and 380 (96%) of participants placed the newborn immediately on the mother’s abdomen after delivery, respectively. The majority of participants, 381 (96.2%), 374 (94.4%), and 383 (96.7%) were dried and stimulated neonates within 30 seconds, respectively. In this study, the commonest parameter frequently practiced by obstetric care providers was weighing the newborn within 90 minutes, recording all care given, and classifying the baby at 396 (100%).

Assessing breathing and color after 30 seconds up to 1 minute, 381 (96.2%), 380 (96%), and 376 (94.9%), covering the baby, including the head, with a clean towel and blanket, 385 (97.2%), 386 (97.5%), and 385 (97.2%), initiating breastfeeding immediately within 1 hour, 393 (99.2%), 389 (98.2%), and 388 (98%) and documenting all cares given on the card chart, 393 (99.2%), 385 (97.2%), and 384 (97%) were the most commonly performed tasks among obstetric care providers, respectively.

On the other hand, applying chlorhexidine gel (4%) to the cord within 30 minutes of delivery, 35 (8.8%), 38 (9.6%), and 34 (8.6%), placing the baby identification bands on the wrist or ankle (within 90 minutes), 17 (4.3%), 38 (9.6%), and 17 (4.3%), and checking the infant’s vital signs, 97 (24.5%), 128 (32.3%), and 100 (25.3%), respectively, were the least performed tasks among obstetric care providers (Table 4).

**Table 4:** Level of ENC practice on three round observations among obstetric care providers in the public hospitals of Gamo, Gofa and Wolayta zones, southern Ethiopia, 2022 (N = 396).

S. N	Variables		Observation 1		Observation 2		Observation 3	
			N	%	N	%	N	%
1	Cloths to dry and warm blankets to cover the infant are ready	No	37	9.3	101	25.5	73	19.7
		Yes	359	90.7	295	74.5	318	80.3
2	The newborn is Placed immediately on the mother’s abdomen	No	13	3.3	19	4.8	16	4
		Yes	383	96.7	377	95.2	380	96
3	Dry and Stimulate neonate within 30 seconds	No	15	3.8	22	5.6	13	3.3
		Yes	381	96.2	374	94.4	383	96.7
4	Assess Breathing and color after 30 seconds up to 1 minute	No	15	3.8	16	4	20	5.1
		Yes	381	96.2	380	96	376	94.9
5	Routine mouth and nose suctioning is not performed	No	64	16.2	104	26.3	90	22.7
		Yes	332	83.8	292	73.7	306	77.3
6	Clamp/tie and cut the cord within 1–3 minutes	No	13	3.3	32	8.1	20	5.1
		Yes	383	96.7	364	91.9	376	94.9
7	Serial instruments are used to clamp and cut the cord	No	2	0.5	17	4.3	7	1.8
		Yes	394	99.5	379	95.7	389	98.2
8	If < 30 breaths per minute, blue tongue, lips or trunk or if gasping then start resuscitating							
		Yes	41	100	55	100	19	100
9	A clean and pre-warm surface is provided for resuscitation	No			10	18.6		
		Yes	41	100	45	81.4	19	100
10	Apply Chlorhexidine gel (4%) on the cord within 30min of delivery	No	361	91.2	358	90.4	362	91.4
		Yes	35	8.8	38	9.6	34	8.6
11	Place the infant in skin-to-skin contact on the mother’s chest	No	49	12.4	75	18.9	87	22
		Yes	347	87.6	321	81.1	309	78
12	Cover the baby including the head with clean towel and blanket	No	11	2.8	10	2.5	11	2.8
		Yes	385	97.2	386	97.5	385	97.2
13	Initiate breastfeeding immediately within 1 hour	No	3	0.8	7	1.8	8	2
		Yes	393	99.2	389	98.2	388	98
14	Apply Tetracycline eye ointment within 90 min of delivery	No	16	4	23	5,8	13	3.3
		Yes	380	96	373	94.2	383	96.7
15	Give Vitamin K, 1mg (for term baby) & 0.5mg (for preterm baby) within 90min	No	31	7.8	39	9.8	30	7.6
		Yes	365	92.2	357	90.2	366	92.4
16	Place the baby identification bands on the wrist or ankle within 90 min	No	379	95.7	358	90.4	379	95.7
		Yes	17	4.3	38	9.6	17	4.3
17	Weigh the Newborn within 90 min, record all care given & classify	Yes	396	100	396	100	396	100
18	The infant vital signs are checked and recorded (at 30 minutes and at 2 hours)	No	299	75.5	268	67.7	296	74.7
		Yes	97	24.5	128	32.3	100	25.3
19	Document all care given on card chart	No	3	0.8	11	2.8	12	3
		Yes	393	99.2	385	97.2	384	97

### Factors associated with essential newborn care practice

In bi-variable logistic regression, sex, profession, qualification, interest in working in the delivery room, work experience, having manageable work load, receiving in-service training, having supportive supervision, and having good knowledge of ENC had a p value of ≤0.25 and were candidate variables for multivariable logistic regression.

In multivariable logistic regression analysis, interest in working in the delivery room, having a manageable work load, receiving in-service training, having supportive supervision, and having good knowledge of ENC were significantly associated variables with the practice of essential newborn care.

The odds of good practice of essential newborn care among obstetric care providers who had interest in working in delivery rooms were 3.16 times higher as compared to those who had no interest in working in delivery rooms (AOR = 3.16, 95% CI: 1.71, 5.83). The odds of good practice of essential newborn care among obstetric care providers who had manageable workloads were 2.96 times higher as compared to their counterparts (AOR = 2.96, 95% CI: 1.78, 4.49).

The odds of good practice of essential newborn care among obstetric care providers who had in-service training on essential newborn care were 3.09 times higher as compared to those who had no training on it (AOR = 3.09, 95% CI: 1.75, 5.45). The odds of good practice of essential newborn care among obstetric care providers who had supportive supervision in the past three months were 3.41 times higher to practice essential newborn care than their counterparts (AOR = 3.41, 95% CI: 1.25, 9.24).

Moreover, the odds of good practice of essential newborn care among obstetric care providers who had good knowledge of essential newborn care were 3.04 times higher as compared to those who had poor knowledge of it (AOR = 3.04, 95%CI: 1.89, 4.90) (Table 5).

**Table 5:** Bivariable and multivariable logistic regression analysis to identify factors associated with essential newborn care practice among obstetric care providers in the public hospitals of Gamo, Gofa and Wolayta zones, southern Ethiopia, 2022 (N = 396).

Variable	Practice of ENC		COR (95% CI)	AOR	P–value
	Good	Poor			
Sex					
** **Male	90(61.6%)	56(38.4%)	1	1	
** **Female	122(48.8%)	128(51.2%)	0.59 (0.39,0.89)	0.61(0.34,1.07)	0.088
Profession					
** **Midwifery	186(51.4%)	176(48.6%)	0.32 (0.14,0.73)	0.62(0.23,1.61)	0.329
** **Other	26(76.5%)	8(23.5%)	1	1	
Qualification					
** **Diploma	58(42.3%)	79(57.7%)	1	1	
** **Degree/above	154(59.5%)	105(40.5%)	1.99(1.31,3.04)	1.17(0.67,2.03)	0.565
Interest					
** **Yes	185(58.9%)	129(41.1%)	2.92(1.75,4.87)	3.16(1.71,5.83)*	0.000
** **No	27(32.9%)	55(67.1%)	1	1	
Work experience					
** **<1 year	34(47.2%)	38(52.8%)	1	1	
** **1–3 years	37(42.5%)	50(57.5%)	0.82(0.44,1.55)	0.63(0.29,1.34)	0.235
** **>3 years	141(59.5%)	96(40.5%)	1.64(0.96,2.79)	1.22(0.58,2.55)	0.598
Work load					
** **Yes	100(45.5%)	120(54.5%)	1	1	
** **No	112(63.6%)	64(36.4%)	2.1(1.39,3.15)	2.96(1.78,4.49)*	0.000
Training on ENC					
** **Yes	143(65.9%)	74(34.1%)	3.08(2.04,4.64)	3.09(1.75,5.45)*	0.000
** **No	69(38.5%)	110(61.5%)	1	1	
Supportive supervision					
** **Yes	31(83.8%)	6(16.2%)	5.08(2.06,12.4)	3.41(1.25,9.24)*	0.016
** **No	181(50.4%)	178(49.6%)	1	1	
Knowledge					
** **Good	162(65.3%)	86(34.7%)	3.69(2.40,5.67)	3.04(1.89,4.90)*	0.000
** **Poor	50(33.8%)	98(66.2%)	1	1	

AOR: adjusted odds ratio, CI: confidence interval, COR: crude odds ratio, ENC: essential newborn care, *P<0.05. Hosmer and Lemeshow test = 0.640.

## Discussion

Although there has been a notable decrease in neonatal mortality rate in Ethiopia, the country still faces a significant public health challenge due to poor essential newborn care being given during birth [15]. This study focuses on assessing factors associated with essential newborn care practice among obstetric care providers in the public hospitals of Gamo, Gofa, and Wolayta zones of southern Ethiopia by using observational data.

This study revealed that the overall good practice of essential newborn care in this study setting is 53.5% (95% CI = 49, 58). The magnitude of good essential newborn care practice among obstetric care providers in our study is similar to previous studies conducted in Sidama region of Ethiopia, and central zone health facilities of Tigray, which showed a good ENC practice among obstetric care providers of 56.6% [13] and 52.4% [23] respectively, and in Vietnam, where 53.2% of obstetric care providers practiced good ENC [7].

On the contrary, the magnitude of good ENC practice among obstetric care providers in our study was higher than the studies conducted in Wolayta zone, in Gedio zone, and in Assosa zone of Ethiopia, which found that 44.4%, 24%, and 41.5% of obstetric care providers practiced good ENC respectively [10, 14, 22], and in Khartoum state teaching hospitals, the good practice of ENC among obstetric care providers is 41.1% [8]. Additionally, our magnitude of good ENC practice among obstetric care providers was lower than studies conducted in south Gondar zone, in Afar regional state, and in Awi zone of Ethiopia, which showed that the good practice of ENC among obstetric care providers is 74.8%, 62.7%, 62.7% respectively [11, 12, 20] and in Nigeria, it is 62.9% [9].

This variation in reported magnitude of ENC practice among obstetric care providers might be due to differences in personal characteristics of obstetric care providers, particularly in relation to work experience of delivery services. Our study was carried out among obstetric care providers with a target on those who are assigned to labor and delivery wards and has direct involvement on ENC practice [10, 14, 22]. Previous research had shown that, having work experience in delivery service have a significant association with ENC practice [14]. Additionally, the variation may also be due to tools used to measure the outcome variable and differences in the way outcome variable were measured. Previous studies were used an interviewer-administered questionnaire to assess level of ENC practice and mean score to define outcome variable [12, 20, 22] and this may have overestimated the magnitude. However, in this study, the magnitude of ENC among obstetric care providers was assessed by using observational checklist and good ENC practice was defined less conservatively as if they correctly performed at least 70% or more of the tasks in the checklists.

According to our study, the odds of good practice of ENC among obstetric care providers who had an interest in working in the delivery room were 3.16 times higher as compared to those who had no interest in working in the delivery room. This finding was supported by findings from studies conducted in Sidama region and in north Shoa zone of Ethiopia, which showed that, those obstetric care providers who had an interest in working in the delivery room were 3.53 times and 1.97 times higher to practice ENC than those who had no interest respectively [6, 13]. The possible explanation for this might be that interest by itself can make obstetric care providers to love their job and give more attention to their activities. It can also increase their appeal to their employees [25].

The odds of good practice of ENC among obstetric care providers who had a manageable workload were 2.96 times higher as compared to their counterparts. The finding is comparable with previous studies conducted in Afar regional state [12], which found that those who had no workload were 2.09 times more likely to practice ENC than their counterparts, and in the south Gondar zone [11], which found that those who had a workload were 2.9 times more likely to practice poor ENC than those who had no workload. To explain this having no load on their employees can give them time to practice all necessary care without being harassed by boredom. This again makes them pay attention to each step of care [26].

Having in-service training on ENC was discovered as a predictor of good ENC practice. The odds of good practice of ENC among obstetric care providers who had in-service training on essential newborn care were 3.09 times higher as compared to those who did not receive it. This result was supported by earlier studies carried out in the Awi zone [20], Assosa zone [22], Sidama region [13], Wolayta zone [14], and Gedeo zone [10]. This resemblance may be due to the fact that in-service training can help obstetric care providers become accustomed to new standards and raise their level of expertise [27, 28]. This is also supported by similarities in training guidelines for obstetric care providers on ENC at a national level [2, 29].

Supportive supervision showed a statistically significant association with ENC practice. The odds of good practice of ENC among obstetric care providers who had supportive supervision within the past three months were 3.41 times higher than their counterparts. This study was supported by studies conducted in the north Shoa zone [6] and in the Gedeo zone [10]. This association might be because supportive supervision can make obstetric care providers identify and fill the gaps in their level of practice and create an initiation to being a competent caregiver [30–32].

The odds of good practice of ENC among obstetric care providers who had good knowledge of essential newborn care were 3.04 times higher as compared to those who had poor knowledge of it. This finding was supported by findings from studies conducted in Awi zone [20], in Wolayta zone [14], and the Bench Sheko zone [33]. The possible reason might be that knowledge can make obstetric care providers pay attention to practice by considering its effects. Since it is a prerequisite to skill, it improves the employee’s efficiency [34, 35].

### Strengths and limitations of the study

#### Strengths of the study

This study was institutional-based and used a standard checklist, including evaluating the baby’s vital signs. Direct observation was conducted three times on a single obstetric care provider, and only obstetric care providers who work in the labor ward and have direct contact with ENC were involved, these ensures the accuracy and reliability of data to make the findings more generalizable to the obstetric care providers in the study area.

#### Limitations of the study

Even if different mechanisms, like conducting observation prior to self-administering questions, were tried to minimize the hawthorn effect on this study, they might still be introduced during repeated observation, and only hospitals were included in this study.

Despite these limitations, the findings from this study will contribute to the understanding of the factors positively associated with good practices of essential newborn care in the study area.

## Conclusions and recommendations

The observed level of essential newborn care practices among obstetric care providers underscores the necessity for targeted interventions that stimulate interest in delivery room work, effectively manage workloads, and offer comprehensive training along with supportive supervision. By concentrating on these aspects and enhancing providers’ knowledge, we can significantly improve essential newborn care practices.

Obstetric care providers who had special training on ENC would support the others who had no special training on it, and those obstetric care providers would have a predilection for the ward.

For those who had a high performance on ENC practice, motivation would be given from the organization and case team to have desire and continuum good performance. Since in-service training had a significant effect on the good practice of ENC, public hospitals would focus on developing customized workshops on targeted training programs that focus on those specific competencies where providers struggle to enhance the capacity building of obstetric care providers thereby improving their overall effectiveness in managing obstetric care. To have better services, health facilities would try to create a fatigue-free working environment. Accountable bodies from the zonal health department and regional health bureau who have up-to-date knowledge and expertise on ENC would adopt all health facilities with a strong and ongoing supportive supervisory strategy. The areas that obstetric care providers need to fill in would be the focus of their supervision, and further study would be conducted by incorporating health centers and other non-governmental health care facilities while considering identifying maternal-related factors.

## Supporting information

S1 File Questionnaire for the study of factors associated with essential newborn care practice among obstetric care providers in public hospitals in Gamo, Gofa, and Wolayta zones, southern Ethiopia. (DOCX)

## List of abbreviations

AOR: Adjusted Odds Ratio
CI: Confidence Interval
ENC: Essential Newborn Care
