# Supplementary material for: Factors associated with essential newborn care practice among obstetric care providers in public hospitals in Gamo, Gofa, and Wolayta zones, southern Ethiopia: A facility-based cross-sectional study, 2022
Source: PLoS One. 2024 Dec 27;19(12):e0314767. doi: 10.1371/journal.pone.0314767 (PMC11676525; doi:10.1371/journal.pone.0314767)
Supplement: S1 File — (DOCX) [file pone.0314767.s001.docx]

# ANNEXES

**English version**

**Annex –I: English Version Questionnaire**

**Part- 1: socio-demographic characteristics of the respondents**

Instruction: Read each items and **circle** your answers for multiple answer questions and fill in the blank space for the dash questions.

| Code | Variables | Response |
| --- | --- | --- |
| 101 | Your sex | 1. Male 2. Female |
| 102 | Age in years | ……………………………… |
| 103 | Your marital status | 1. Unmarried  2. Married  3. Widowed  4. Divorced |
| 104 | What religion you belong? | 1. Muslim 2. Orthodox  3. Protestant 4. Catholic  5. Others |
| 105 | What is your profession (field of study)? | …………………………………………………. |
| 106 | What is your qualification? | 1. Diploma  2. Degree  3. Others (specifiy)…………………….. |
| 107 | Your monthly salary | …………………………..in ETH. Birr |
| 108 | What is your work experience? | ………………… in year |

**Part-II: personal related questions**

| Code | Variables | Response |
| --- | --- | --- |
| 201 | Did you interested on working in delivery room? | 1. Yes 2. No |
| 202 | What is your work experience of delivery services? | -------------------in years |
| 203 | For how many cases did you give care per day? | ------------------- in number |
| 204 | Did you ever before receive in-service training on Essential newborn care? | 1. Yes  2. No |
| 205 | If yes for Q204, how many times have you got  In-service training? | ------------------ in number |
| 206 | Did you have supportive supervision with in the last three months? | 1. Yes 2. No |
| 207 | If yes for **Q206,** how many times have you got supportive supervision? | -------------- in number |
| **Part III: Institutional related questions**   \| 301 \| What is your facility type? \| 1. Primary hospital 2. General hospital 3. Teaching/Referral hospital \| \| --- \| --- \| --- \| \| 302 \| Have you ever had difficulty giving essential newborn care due to lack of training guidelines at your facility with in the last three months? \| 1= Yes 0= No \| \| 303 \| Have you ever had difficulty giving cord care due to a lack of cord tie at your facility with in the last three months? \| 1= Yes 0= No \| \| 304 \| Have you ever had difficulty giving a baby’s airway due to a lack of suction device at your facility with in the last three months? \| 1= Yes 0= No \| \| 305 \| Have you ever miss giving vitamin k due to its absence at your facility with in the last three months? \| 1= Yes 0= No \| \| 306 \| Have you ever miss giving TTC eye ointment due to its absence at your facility with in the last three months? \| 1= Yes 0= No \| \| 307 \| Have you ever had difficulty to put a baby’s identification band due to its absence at your facility with in the last three months? \| 1= Yes 0= No \| \| 308 \| Have you ever had difficulty giving a baby’s airway due to a lack of term and premature size masks at your facility with in the last three months? \| 1= Yes 0= No \| | | |

**Part-IV: knowledge questions regarding specific Essential Newborn Care**

**Instruction**-Read each items and **circles** your answers for each question. More than **one response** is possible if you have more than one answer for one item of question.

| Code | Variables | Response |
| --- | --- | --- |
| 401 | When do essential newborn cares should start? | 1. Before birth 2. During birth  3. After birth 4. I don’t know |
| 402 | What method we can use to prevent infection in newborn? | 1. Proper hand washing 2. Using appropriate personal protective equipment 3. Maintaining a clean environment 4. Other (specify) -------------------- |
| 403 | Where newborn should kept immediately after birth? | 1. Beside the mother  2. With someone else  3. On the mother’s chest/ belly  4. On newborn bed /table  5. Other (specify)---------------- |
| 404 | What method we can use to prevent hypothermia in newborn? | 1. Immediately drying  2. Allowing skin to skin contact  3. Early bathing  4. Other( specify)-------------- |
| 405 | When do newborn should be bathed after delivery? | 1. Immediately  2.Within the first 24 hours of delivery  3. After 24 hours of delivery  4. Other (specify)---------------- |
| 406 | How often do you take the infants vital sign including temperature? | 1. Only once within 6 hours 2. At least twice in the first 2 hours 3. Every 15 minutes for the first 2 hours 4. No need of temperature measure |
| 407 | What do you do if the baby **no**t cries immediately after birth? | 1. Cover the baby and allow skin to skin contact  2. Call a help and start resuscitation  3. Put bay on newborn table and give mother care  4. Other (specify)------------------- |
| 408 | If a baby is not breathing well after drying, clearing the air way and rubbing the back once or twice, what you should give? | 1. More stimulation to breath  2. Ventilation with bag and mask  3. Place on oxygen  4. Other (specify)-------------- |
| 409 | When to start resuscitation in a newborn | 1. Not breathing or ˂40 breaths/mn 2. Not breathing or ˂30breaths/mn 3. Not breathing or ˂20 breaths/mn 4. Not breathing or ˂50breaths/mn |
| 410 | In which position the baby’s head should be put to help open the airway? | 1. A flexed position of the head 2. The slightly extended position of head 3. Hyperextended position of head 4. Other (specify)…………….. |
| 411 | During ventilation of new born, what is the recommended breath per minutes? | 1. 30 breaths per minute  2. 40 breaths per minutes  3. 60 breaths per minute |
| 412 | When we should initiate breast feeding for new born baby? | 1. After 6 hours of delivery  2. Within 1-6 hours of delivery  3. Within the first hour of delivery  4. Other (specify)---------------- |
| 413 | How long should a mother exclusively breast feed her child? | 1. Less than 6 month  2. For 6 months  3. Greater 6 months  4. Other( specify)------------------ |
| 414 | How long should you wait to clamp or tie the umbilical cord of a crying baby? | 1. Clamp or tie Immediately  2. Clamp or tie 1-2 minutes of delivery  3. Clamp or tie 2-3 minutes of delivery/after pulsation of umbilical artery stopped |
| 415 | What is the recommended treatment of eye infection in newborn? | 1. Apply nothing  2. Apply breast milk in the eye  3. Clean eye with sterile water  4. Apply silver nitrate/tetracycline |
| 416 | What is the recommended action to prevent bleeding in newborn? | 1. Breastfeed the child  2. Not necessary to give anything  3. Give vitamin K  4. Other (specify)----------- |
| 417 | How do you define low birth weight? It is a weight of …….. | 1. <3000gm 2. <2500gm  3. <1500 gm 4. <1000 gm |
| 418 | The recommended care for Low Birth Weight baby? | 1. Bath often  2. Breast feeding early and frequently  3. Keep the child warm  4. Prevent from developing infection |
| 419 | The recommended dose of vit k for preterm baby is? | 1. 1mg 2. 0.5mg  3. 2gm 4. Other (specify)----------- |
| 420 | What is the best timing for first postnatal visit? | 1. Within the first 24 hours of delivery  2. On the 3rd day of delivery  3. On the 7th day of delivery  4. Other(specify)----------------- |
| 421 | What vaccination is recommended to give to neonate at time of birth? | 1. HepB 3. BCG 2. OPV-0 4. OPV-1 |

**Part- V: Clinical observational checklist to assess practice of essential newborn care**

**Instruction**: Please collect the information by observing the treatment and care of babies with the relevant condition.

**Directions:** Rate the performance of each step or task using the following rating scale:

**Scoring system**: each participant is evaluated for three tasks or number of newborn cares to reach an overall score. Number 1 and 0 are assigned to indicate whether the task is performed or not.

1= if procedure done correctly. 0= if procedure is not done correctly or missed.

| Code | Variables | Scores | | | | | | | |
| --- | --- | --- | --- | --- | --- | --- | --- | --- | --- |
|  |  | 1 | 0 |  | 1 | 0 |  | 1 | 0 |
| 501 | Cloths to dry and warm blankets to cover the infant are ready |  |  |  |  |  |  |  |  |
| 502 | The newborn is Placed immediately on the mother’s abdomen after a vaginal delivery. |  |  |  |  |  |  |  |  |
| 503 | Dry and Stimulate neonate within 30 seconds |  |  |  |  |  |  |  |  |
| 504 | Assess Breathing and color after 30 seconds up to 1 minute |  |  |  |  |  |  |  |  |
| 505 | Routine mouth and nose suctioning is not performed |  |  |  |  |  |  |  |  |
| 506 | If the baby does cry or breaths well clamp/tie and cut the cord within 1-3 minutes |  |  |  |  |  |  |  |  |
| 507 | Serial instruments are used to clamp and cut the cord |  |  |  |  |  |  |  |  |
| 508 | If < 30 breaths per minute, blue tongue, lips or trunk or if gasping then start resuscitating |  |  |  |  |  |  |  |  |
| 509 | A clean and pre-warm surface is provided for resuscitation |  |  |  |  |  |  |  |  |
| 510 | Apply Chlorhexidine gel (4%) on the cord within 30min of delivery |  |  |  |  |  |  |  |  |
| 511 | Place the infant in skin-to-skin contact on the mother’s chest |  |  |  |  |  |  |  |  |
| 512 | Cover the baby including the head with clean towel and blanket while the baby is on mother’s abdomen as required. |  |  |  |  |  |  |  |  |
| 513 | Initiate breastfeeding immediately within 1 hour |  |  |  |  |  |  |  |  |
| 514 | Apply Tetracycline eye ointment within 90 min of delivery |  |  |  |  |  |  |  |  |
| 515 | Give Vitamin K, 1mg (for term baby) and 0.5mg (for preterm baby) IM on anterior mid-lateral thigh (within 90min) |  |  |  |  |  |  |  |  |
| 516 | Place the baby identification bands on the wrist or ankle within 90 min |  |  |  |  |  |  |  |  |
| 517 | Weigh the Newborn within 90 min, record all care given and classify the baby. |  |  |  |  |  |  |  |  |
| 518 | The infant vital signs are checked and recorded in a specific part of the mother file or newborn file, if existing (at 30 minutes and at 2 hours) |  |  |  |  |  |  |  |  |
| 519 | Document all care given on card chart |  |  |  |  |  |  |  |  |
